# The value of daily platelet counts for predicting dengue shock syndrome: Results from a prospective observational study of 2301 Vietnamese children with dengue

**DOI:** 10.1371/journal.pntd.0005498

**Published:** 2017-04-27

**Authors:** Phung Khanh Lam, Tran Van Ngoc, Truong Thi Thu Thuy, Nguyen Thi Hong Van, Tran Thi Nhu Thuy, Dong Thi Hoai Tam, Nguyen Minh Dung, Nguyen Thi Hanh Tien, Nguyen Tan Thanh Kieu, Cameron Simmons, Bridget Wills, Marcel Wolbers

**Affiliations:** 1 Oxford University Clinical Research Unit, Hospital for Tropical Diseases, Ho Chi Minh City, Viet Nam; 2 Hospital for Tropical Diseases, Ho Chi Minh City, Viet Nam; 3 Department of Microbiology and Immunology, The Peter Doherty Institute, University of Melbourne, Australia; 4 Centre for Tropical Medicine and Global health, Nuffield Department of Clinical Medicine, University of Oxford, United Kingdom; Oregon Health and Science University, UNITED STATES

## Abstract

**Background:**

Dengue is the most important mosquito-borne viral infection to affect humans. Although it usually manifests as a self-limited febrile illness, complications may occur as the fever subsides. A systemic vascular leak syndrome that sometimes progresses to life-threatening hypovolaemic shock is the most serious complication seen in children, typically accompanied by haemoconcentration and thrombocytopenia. Robust evidence on risk factors, especially features present early in the illness course, for progression to dengue shock syndrome (DSS) is lacking. Moreover, the potential value of incorporating serial haematocrit and platelet measurements in prediction models has never been assessed.

**Methodology/Principal findings:**

We analyzed data from a prospective observational study of Vietnamese children aged 5–15 years admitted with clinically suspected dengue to the Hospital for Tropical Diseases in Ho Chi Minh City between 2001 and 2009. The analysis population comprised all children with laboratory-confirmed dengue enrolled between days 1–4 of illness. Logistic regression was the main statistical model for all univariate and multivariable analyses. The prognostic value of daily haematocrit levels and platelet counts were assessed using graphs and separate regression models fitted on each day of illness. Among the 2301 children included in the analysis, 143 (6%) progressed to DSS. Significant baseline risk factors for DSS included a history of vomiting, higher temperature, a palpable liver, and a lower platelet count. Prediction models that included serial daily platelet counts demonstrated better ability to discriminate patients who developed DSS from others, than models based on enrolment information only. However inclusion of daily haematocrit values did not improve prediction of DSS.

**Conclusions/Significance:**

Daily monitoring of platelet counts is important to help identify patients at high risk of DSS. Development of dynamic prediction models that incorporate signs, symptoms, and daily laboratory measurements, could improve DSS prediction and thereby reduce the burden on health services in endemic areas.

## Author summary

Dengue is a very common, potentially serious, mosquito-borne viral infection. The spectrum of clinical disease is broad. Dengue shock syndrome (DSS), seen primarily in children, is the most serious life-threatening manifestation. Early identification of children presenting with dengue who are likely to develop DSS could improve triage and resource allocation in endemic areas. This study, based on data from 2301 Vietnamese children hospitalized with dengue, aimed to assess the value of readily available clinical and laboratory markers, especially platelet counts and haematocrit levels, in predicting DSS. In addition to risk factors present at the first assessment within 1–4 days from fever onset (vomiting, higher temperature, palpable liver, lower platelet count), we showed that serial daily platelet counts provide useful additional information to identify at an early stage children who are likely to develop shock. Although absolute platelet values were already known to be important, this is the first study to confirm the usefulness of sequential daily platelet counts. It also provides proof of concept for the value of incorporating serial laboratory and clinical signs into future dynamic prognostic models to allow for earlier identification and better management of children at risk of DSS.

## Introduction

Dengue is the most important mosquito-borne viral infection affecting humans. Incidence has increased dramatically over the last 50 years, with approximately 100 million symptomatic dengue infections now estimated to occur annually across more than 100 countries [1,2]. Although the majority of symptomatic infections manifest as a non-specific self-limited febrile illness, a small proportion of patients progress to more severe and occasionally life-threatening disease. Of particular concern is a vasculopathy characterized by endothelial dysfunction and plasma leakage that becomes apparent several days into the illness, often around the time of defervescence; this phenomenon tends to be more pronounced in children, and may be severe enough to cause hypovolaemic shock, i.e. dengue shock syndrome (DSS) [3,4]. Other associated phenomena include a) thrombocytopenia and a coagulopathy that may result in severe bleeding, and b) organ involvement (e.g. hepatitis) that occasionally progresses to major organ failure.

As yet no effective anti-viral or immunomodulatory therapy has been identified [5], but with careful observation and judicious use of intravenous fluid therapy to counteract plasma leakage, most notably urgent volume resuscitation for patients with established DSS, mortality rates have been reduced to less than 1% in specialist centres [6]. However, mortality rates up to 10% are still occasionally reported [7,8]. One contributing factor is that early identification of individuals likely to progress to severe disease is difficult. Consequently large numbers of patients with possible dengue are admitted to hospitals in dengue-endemic areas primarily for observation, overburdening the healthcare systems such that limited local resources are not used to maximal advantage for the small proportion of patients who do need expert care.

Effective triage to identify high-risk patients early in the illness course should make it possible to improve patient referral strategies to high dependency or intensive care units (HDU/ICUs), a crucial element in maximising the efficiency of healthcare utilisation in low to middle income countries with limited resources. World Health Organization (WHO) guidelines for diagnosis and management of dengue now specify a number of warnings signs for likely progression to severe disease, but these are based largely on expert opinion and still require validation in well-designed studies [9,10]. Moreover, in practice, physicians often rely on their own clinical experience rather than following WHO guidelines [11]. Identification of objective and evidence-based risk factors, coupled with development of practical tools such as clinical prediction models for severe dengue, could prove very useful to reduce the number of unnecessary hospital admissions, and facilitate HDU/ICU referrals to target care to the patients in greatest need.

Several groups have tried to identify risk factors and develop prediction models for DSS [12–19]. Most of these studies were carried out in children [14,16,17,19], although some focused on or included adults [12,13,15,18], who may have different underlying pathophysiology compared to children. However, only a few studies were prospective [17–19], and the number of patients who developed severe disease in these studies was low, ranging from 10 to 55 patients. Furthermore, in all cases data on risk factors were only collected at baseline, i.e. the time of presentation, hospital admission, diagnosis, or initiation of an intervention. Predictions based on baseline risk factors are only valid at a single time-point and tend to become less relevant as a disease progresses [20]. Where longitudinal data are available at intervals during the course of an illness, they present an opportunity to improve predictions by updating the models using sequential information.

In this study, we used a comprehensive prospective dataset collected from 2301 children hospitalized during the febrile phase of dengue at the Hospital for Tropical Diseases in Ho Chi Minh City, in order to assess potential risk factors for progression to DSS. We assessed the predictive values of clinical and laboratory candidate risk factors collected at study enrolment, focusing on parameters that are usually available in dengue-endemic countries like Vietnam. In addition we investigated whether longitudinal platelet values and haematocrit levels measured daily during hospitalization might improve prediction of DSS.

## Methods

### Ethics statement

The study was approved by the Scientific and Ethical Committee of Hospital for Tropical Diseases (HTD) and the Oxford Tropical Research Ethics Committee.

### Study population and participants

A prospective observational study of children hospitalized with suspected dengue at the HTD in Ho Chi Minh City, Viet Nam, was conducted between 2001 and 2009. The cohort included any child aged between 5 and 15 years admitted to the paediatric dengue ward at HTD with clinically suspected dengue, whose parent/guardian gave written informed consent for them to be enrolled in the study following detailed explanation by a trained study doctor. Consecutive suspected dengue cases identified during the morning ward round were approached by study staff as potential participants; commencing on Monday morning the process continued until up to 10 suspected dengue cases had been enrolled for that week. Of note, the paediatric dengue ward is responsible for managing children with uncomplicated illness only, and HTD policy dictates that any child who develops DSS or about whom there is concern (typically development of warning signs necessitating monitoring more frequently than 4–6 hourly) is transferred to the Paediatric Intensive Care Unit (PICU). During the study period all children admitted to PICU with DSS were recruited into a concurrent pediatric cohort [6,21].

After enrolment, baseline information including demographic characteristics, clinical history, and examination findings was documented using a structured case report form. The study doctors then followed all patients daily throughout the hospitalization and recorded information on therapeutic interventions, supportive care and major clinical events, in particular the need for transfer to PICU. For those patients who were transferred to PICU additional data was obtained from the case report forms that were completed as part of the PICU DSS study [6]. All patients were asked to return for follow-up assessments one month later. Venous blood samples for dengue diagnostics (see below) were obtained on the day of enrolment, the day of discharge or defervescence, and at the follow up visit. In addition, small volume daily blood samples were obtained as part of standard care for dengue patients, to measure haematocrit levels and platelet counts. Clinical management was in accordance with the HTD dengue treatment guidelines, which were based on the WHO 1997 dengue guidelines [22], and recommendations from the Vietnamese Ministry of Health. There were no substantive changes to the HTD treatment guidelines during the study, and the same group of senior clinicians was responsible for supervision of dengue patient management throughout the whole study period.

The study population for this analysis comprised participants enrolled in the observational study who were subsequently confirmed to have dengue. As DSS occurs most frequently on day 5 or 6 of illness [6], the main analysis was restricted to patients who were enrolled between days 1–4 of illness (i.e. on the day of fever onset (day 1) or the subsequent 3 days) and who did not experience DSS on the day of enrolment.

### Dengue diagnostics

RT-PCR was performed on the enrolment specimen using established methodology [23,24]. Dengue IgM and IgG capture ELISAs were performed on paired enrolment and early convalescent specimens. During the study period the diagnostic laboratory used a number of different serological tests, following the manufacturer's instructions for commercial kits (Dengue Duo IgM and IgG Capture ELISA, PanBio, Australia), or established standard operating procedures for in-house methods [25].

Laboratory-confirmed dengue was defined by detection of DENV-RNA in plasma by RT-PCR, or by seroconversion on the capture ELISA. A number of different methods to classify primary/secondary immune status are recommended, but all have limitations [26–28]. Given the range of sero-diagnostic tests employed during the 9 years of the study, we elected to use a simple, pragmatic immunologic classification based on capture IgG results only: a probable primary infection was defined by negative dengue-specific IgG results on acute and early convalescent plasma, at least one specimen being obtained during the second week of illness, and a probable secondary infection by a positive dengue-specific IgG identified on either or both the acute and early convalescent specimens. All other cases were considered unclassifiable, in general due to the absence of a suitable specimen at the appropriate time-points.

### Clinical outcome and candidate predictors

The outcome of interest was DSS, defined as development of narrow pulse pressure (≤ 20 mmHg) or hypotension for age with evidence of impaired peripheral perfusion [10].

Table S1 in S1 Appendix describes the pre-defined candidate predictors assessed at the time of enrolment in the study (baseline candidate predictors). These predictors included the presence of WHO warning signs [10], as well as other clinical signs and symptoms that have been identified as risk factors for severe dengue in other studies [29,30]. As dengue serotype and immune status were missing for a number of participants, and since this information would not normally be available to the treating physician in routine clinical practice, these parameters were only included in univariate analyses but not in the multivariable analysis. Finally, we wished to take into account the evolving nature of acute dengue over several days in the analysis; we defined the day of illness as the number of days up to and including the day of interest, with the day of fever onset defined as day 1 of illness.

The platelet count and haematocrit level have long been considered crucial factors to monitor in patients with dengue, but their role as potential predictors of progression to severe disease has rarely been formally investigated [29,30]. Using the sequential daily haematocrit and platelet values available for the study participants we were particularly interested to investigate relationships between aspects of the platelet and/or haematocrit dynamics (baseline value, current value, percentage (%) change from the previous day) with development of DSS.

### Statistical analysis

Logistic regression was the main statistical model for all univariate and multivariable analyses. The development and validation of a prediction model for the development of DSS using baseline covariates followed current standard methodology and recommendations for prognostic modeling [31,32], and multiple imputation [33]. Modeling assumptions of logistic regression (linearity and additivity) were carefully assessed and the final model was simplified using stepwise backwards model selection. Model performance was summarized using the Brier score, the area under the receiver-operating curve (AUC) and calibration measures. All performance measures were corrected for potential over-fitting by 10-times 10-fold cross-validation. Details on model development, variable selection, alternative models, and validation are provided in the S1 Appendix.

We investigated the value of daily haematocrit levels and platelet counts to predict subsequent DSS using both graphical and regression methods. For these analyses we included only patients enrolled on day 3 of illness, to avoid potential confounding effects of other time-varying signs and symptoms that were only assessed at baseline, on outcome. For regression analysis, we fitted separate logistic regression models with the occurrence of DSS as the outcome, and different aspects of haematocrit and platelet dynamics (baseline value, current value, or % change from previous day) as covariates for each day of illness using only patients who were still at risk on that day (i.e. those without DSS up to and including that day). In addition, these models were adjusted for all baseline covariates (other than the platelet count) selected by the final baseline model. The performance of these models was summarized in terms of the AUC and was corrected for optimism using cross-validation.

We imputed missing predictor values by multiple imputation using the MICE algorithm [34], resulting in 20 imputed datasets (see Additional file 1 for a detailed description). All analyses involving baseline risk factors used these imputed datasets, except for univariate analyses that were based on complete-case analyses. We also verified results from analyses based on multiple imputation against results from complete-case analyses [35]. For simplicity, all analyses of longitudinal platelet counts were based on available data only, without imputation. All analyses were performed with the statistical software R version 3.2.0 (2015-04-16) [36] and its companion packages [34,37–41].

## Results

### General description of study participants

**Fig 1:**
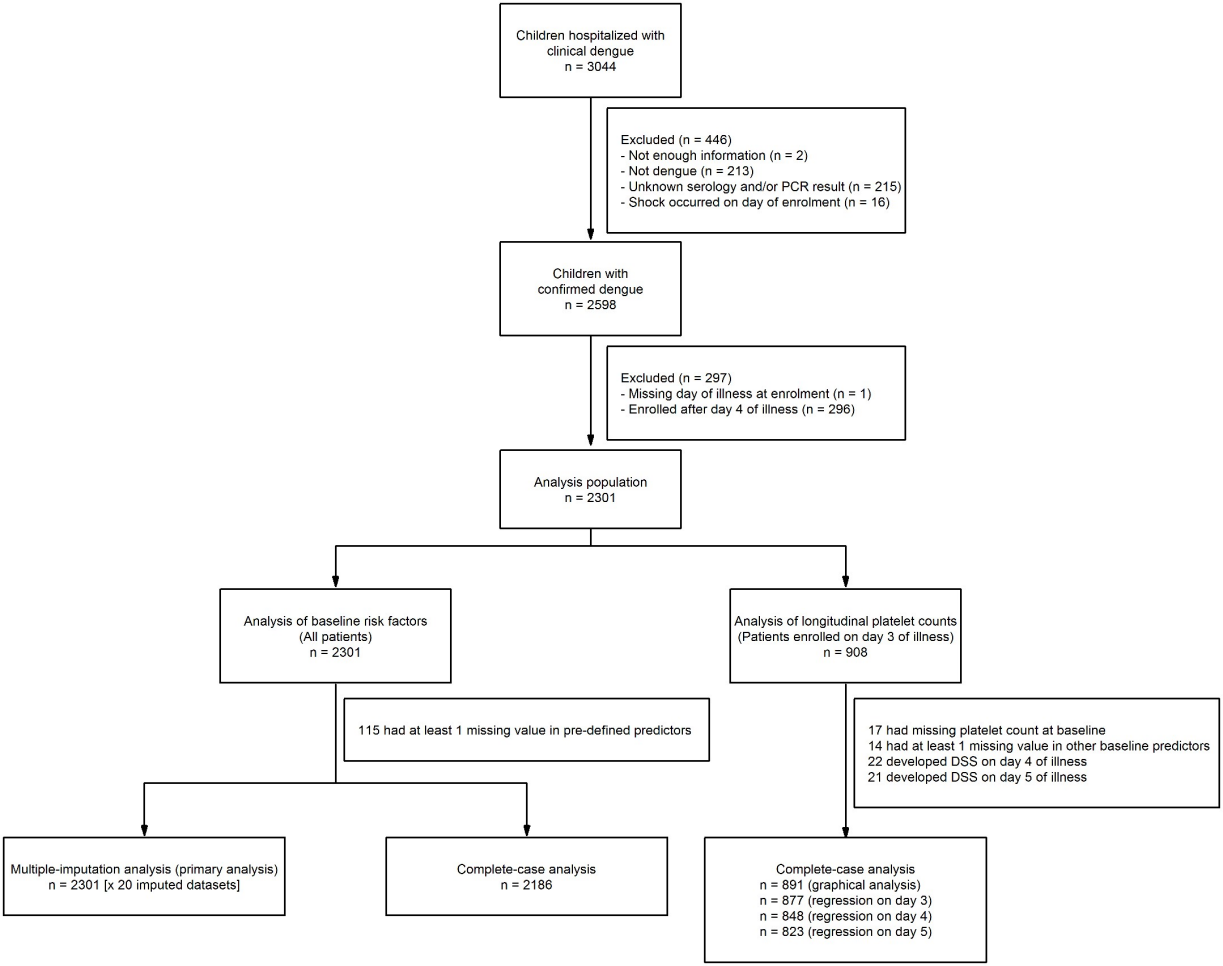
Participant flow diagram.

A total of 3044 children admitted to the paediatric dengue ward between 2001 and 2009 were enrolled in the observational study, of whom 2598 had laboratory confirmed dengue. Among these participants, 2301 were enrolled between days 1 and 4 of illness and formed the main analysis population for evaluating baseline risk factors. For investigating longitudinal haematocrit levels and platelet counts, the analysis was restricted to the 908 patients enrolled on day 3 of illness (see Fig 1). The main outcome of interest (development of DSS or not) was available for all patients but 115/2301 (5%) of participants had at least one missing candidate predictor.

Table 1 summarizes the characteristics of these 2301 study participants at enrolment. More males than females were enrolled in the study and the median age was 12 years (interquartile range (IQR) 10–13 years). Most patients were still febrile at enrolment, with 96% of the participants having a temperature ≥38°C. Haemodynamic parameters, including pulse rate and systolic blood pressure, were within the normal range, apart from one child with known congenital heart disease. At the time of enrolment, platelet values were already beginning to fall (median 134,000, IQR 97,000–178,000 cells/mm^3^), whereas haematocrit values remained consistent with expected normal values for age (median 40%, IQR 37–42%). Among the 2152/2301 (94%) cases for whom RT-PCR was performed, most patients were infected with DENV-1 (956/2152, 44%) or DENV-2 (553/2152, 26%), with considerably lower representation for DENV-3 and DENV-4. Among cases whose serology status could be assessed, 94% (1938/2053) had probable secondary infections.

**Table 1:** Patient characteristics at enrolment, and outcomes during hospitalization (N = 2301).

Characteristic	n	Summary statistic
Age [year]	2300	12 (10, 13)
Sex: Female	2301	939 (41%)
Weight [kg]	2296	34 (27, 42)
Day of illness at enrolment	2301	3 (3, 4)
History of tiredness: Yes	2298	1936 (84%)
History of vomiting: Yes	2295	832 (36%)
Tourniquet test	2289	
- Negative		1164 (51%)
- Equivocal		458 (20%)
- Positive		667 (29%)
Temperature [°C]	2299	39 (38, 40)
Pulse [beats/min]	2297	100 (100, 116)
Systolic blood pressure [mmHg]	2298	92 (90, 100)
Mucosal bleeding: Yes	2283	158 (7%)
Abdominal pain: Yes	2291	465 (20%)
Palpable liver: Yes	2279	217 (10%)
Haematocrit [%]	2259	40 (37, 42)
Platelet count [cells/mm^3^]	2258	134,000 (97,000, 178,000)
Serotype	2152	
- DENV-1		956 (44%)
- DENV-2		553 (26%)
- DENV-3		195 (9%)
- DENV-4		169 (8%)
- Mixed		8 (<1%)
- Negative		271 (13%)
Immune status	2301	
- Probable secondary		1938 (84%)
- Probable primary		115 (5%)
- Inconclusive		248 (11%)
Referred to PICU: Yes	2301	179 (8%)
DSS: Yes	2301	143 (6%)
Day of illness at shock	143	
- 3		2 (1%)
- 4		33 (23%)
- 5		70 (49%)
- 6		28 (20%)
- 7 or 8		10 (7%)
Bleeding during hospitalization	2288	
- None		1333 (58%)
- Skin only		843 (37%)
- Mucosal		112 (5%)
Platelet nadir [cells/mm³]	2279	65,200 (41,000, 99,000)
Day of illness at platelet nadir	2279	6 (5, 7)
Maximum haematocrit [%]	2283	44 (41, 47)
Day of illness at maximum haematocrit	2283	5 (4, 6)
Overall haemoconcentration [%]	2282	13 (6, 22)

Summary statistics are median (interquartile range) for continuous variables and frequency (%) for categorical variables.

Platelet nadir and maximum haematocrit (HCT) were, respectively, the minimum platelet count and the maximum HCT value observed between days 3 and 8 of illness. Overall haemoconcentration was the percentage change of the maximum HCT compared to the normal HCT for a specific patient. The normal HCT was the HCT value at follow-up (after day 14 of illness), or the minimum HCT value before day 2 of illness (provided the platelet count at the same time was ≥200,000 cells/mm^3^), or the population value taken from a dataset including >1000 healthy Vietnamese children (37% for children from 5–10 years old, 38.5% for females >10 years old, 40% for males >10 years old).

PICU: Paediatric Intensive Care Unit, DSS: Dengue Shock Syndrome.

During hospitalization, 179/2301 cases (8%) were referred to PICU for more intense monitoring and management (Table 1), with 143/2301 (6%) developing DSS. All the DSS cases recovered following appropriate resuscitation. The remaining 36 patients were transferred to PICU due to concerns about warning signs, but all recovered fully without further disease progression. There was no systematic time trend in the incidence of DSS over the study period (linear trend test: p = 0.35). Although DSS was identified on all illness days from 3 to 8, 92% of the cases occurred between days 4 and 6.

New bleeding was observed after hospitalisation in 955/2288 (42%) cases, but only a small number of patients (112/955 (12%)) experienced mucosal bleeding. The most frequent mucosal bleeding sites were nose (58/112) and gum (34/112), with less frequent sites being gastrointestinal (15/112) and vaginal (7/112). No case was considered severe enough to warrant blood transfusion. The platelet nadir commonly occurred around day 6 of illness with a median nadir of 65,200 (IQR: 41,000–99,000) cells/mm^3^. In many patients the maximum haematocrit value was recorded on the same day, with a median level of 44% (IQR 41–47%) and a corresponding median maximum haemoconcentration of 13% (IQR 6–22%).

### Baseline risk factors for development of DSS

Univariate and multivariable associations between baseline candidate predictors and the development of DSS are shown in Table 2. Male gender, history of vomiting, palpable liver, higher temperature, and lower platelet count, all assessed at enrolment, were significant risk factors for developing DSS in both univariate and multivariable analyses. Enrolment at an earlier day of illness was associated with a higher risk of developing DSS after adjusting for other covariates although not in the univariate analysis. While none of the probable primary cases developed DSS, 138/1938 (7%) of probable secondary and 5/248 (2%) of the inconclusive cases developed DSS. The multivariable analysis results were consistent between multiple imputation and complete-case analyses (Table S3 in S1 Appendix).

**Table 2:** Unadjusted and adjusted effects of candidate predictors at enrolment on clinical outcome (N = 2301).

Predictor		No DSS (N = 2158)		DSS (N = 143)	Unadjusted effect			Adjusted effect		
	n	Summary statistic	n	Summary statistic	OR*	(95% CI)	p	OR*	(95% CI)	p
Age [year]	2157	12 (10, 13)	143	11 (10, 13)	0.97	(0.90, 1.05)	0.41	0.94	(0.85, 1.04)	0.22
Sex: Female	2158	892 (41%)	143	47 (33%)	0.69	(0.48, 0.99)	0.04	0.65	(0.44, 0.94)	0.02
Weight [kg]	2153	35 (27, 42)	143	33 (27, 40)	0.99	(0.98, 1.01)	0.43	0.99	(0.97, 1.02)	0.62
Day of illness at enrolment	2158	3 (3, 4)	143	3 (3, 4)	0.94	(0.75, 1.18)	0.58	0.68	(0.52, 0.88)	0.004
History of tiredness: Yes	2155	1816 (84%)	143	120 (84%)	0.97	(0.63, 1.58)	0.91	0.88	(0.54, 1.44)	0.62
History of vomiting: Yes	2154	754 (35%)	141	78 (55%)	2.30	(1.63, 3.25)	<0.001	2.20	(1.54, 3.15)	<0.001
Tourniquet test	2149		140				0.90			0.46
- Negative		1095 (51%)		69 (49%)	1.00			1.00		
- Equivocal		428 (20%)		30 (21%)	1.11	(0.71, 1.72)		1.13	(0.71, 1.78)	
- Positive		626 (29%)		41 (29%)	1.04	(0.69, 1.54)		0.83	(0.54, 1.26)	
Temperature [°C]	2157	39 (38, 40)	142	39 (39, 40)	1.33	(1.04, 1.71)	0.02	1.39	(1.07, 1.82)	0.01
Pulse [beats/min]	2155	100 (100, 120)	142	100 (100, 120)	1.07	(0.91, 1.27)	0.40	1.03	(0.86, 1.23)	0.74
Systolic blood pressure [mmHg]	2156	100 (90, 100)	142	90 (90, 100)	1.01	(0.81, 1.23)	0.92	1.00	(0.81, 1.25)	0.96
Mucosal bleeding: Yes	2142	146 (7%)	141	12 (9%)	1.27	(0.65, 2.26)	0.46	1.15	(0.60, 2.20)	0.67
Abdominal pain: Yes	2149	424 (20%)	142	41 (29%)	1.65	(1.12, 2.39)	0.01	1.05	(0.66, 1.68)	0.82
Palpable liver: Yes	2136	189 (9%)	143	28 (20%)	2.51	(1.59, 3.84)	<0.001	1.74	(1.02, 2.96)	0.04
Haematocrit [%]	2120	40 (37, 42)	139	39 (38, 43)	1.03	(0.99, 1.07)	0.18	1.03	(0.98, 1.07)	0.23
Platelet count [cells/mm^3^]	2119	140,000 (100,000, 180,000)	139	100,000 (80,000, 150,000)	0.92	(0.89, 0.95)	<0.001	0.89	(0.86, 0.93)	<0.001
Serotype	2014		138				<0.001	-	-	-
- DENV-1		891 (44%)		65 (47%)	1.00			-	-	-
- DENV-2		507 (25%)		46 (33%)	1.24	(0.84, 1.84)	0.28	-	-	-
- DENV-3		188 (9%)		7 (5%)	0.51	(0.21, 1.06)	0.09	-	-	-
- DENV-4		156 (8%)		13 (9%)	1.14	(0.59, 2.06)	0.67	-	-	-
- Mixed		6 (0%)		2 (1%)	4.57	(0.66, 20.29)	0.07	-	-	-
- Negative		266 (13%)		5 (4%)	0.26	(0.09, 0.59)	0.004	-	-	-
Immune status	2158		143				<0.001	-	-	-
- Probable secondary		1800 (84%)		138 (97%)	1.00			-	-	-
- Probable primary		115 (5%)		0 (0%)	0.06	(0.00, 0.39)	<0.001	-	-	-
- Inconclusive		243 (11%)		5 (3%)	0.29	(0.11, 0.63)	<0.001	-	-	-

Summary statistic is absolute count (%) for categorical variables and median (IQR) for continuous data.

Unadjusted analyses were based on complete cases, adjusted analyses were based on multiple-imputed datasets.

Serotype and immune status were not included in the multivariable analysis.

* For continuous variables, ORs correspond to +1 year (age), +1 kg (weight), +1°C (temperature), +10 beats/min (pulse), +10 mmHg (systolic blood pressure), +1% (haematocrit), +10,000 cell/mm^3^ (platelet count).

OR: Odds Ratio, 95% CI: 95% Confidence Interval, p: p value.

OR, CI and p-value for immune status were calculated based on penalized maximum likelihood (Firth’s correction).

### Baseline prognostic models for development of DSS

Age, sex, day of illness, history of vomiting, temperature, palpable liver and platelet count at enrolment were retained in the logistic regression model with stepwise variable selection based on multiple imputation (Table 3). The same predictors and similar effect sizes were identified in the complete-case analysis (Table S3 in S1 Appendix). The final reduced model had a moderate AUC of 0.70 and good calibration, and its performance was comparable to or better than alternative modeling approaches (Table S4 in S1 Appendix).

**Table 3:** Reduced baseline logistic regression model for development of DSS and its performance (N = 2301).

**Predictor**	**OR**	**(95% CI)**	**p value**
Age [+ 1 year]	0.93	(0.86, 1.01)	0.09
Sex: Female	0.64	(0.44, 0.92)	0.02
Day of illness at enrolment [+ 1 day]	0.69	(0.53, 0.89)	0.005
History of vomiting: Yes	2.20	(1.55, 3.13)	<0.001
Temperature [+ 1°C]	1.36	(1.06, 1.76)	0.02
Palpable liver: Yes	1.76	(1.11, 2.79)	0.02
Platelet count [+ 10,000 cells/mm^3^]	0.89	(0.86, 0.93)	<0.001
**Performance criteria**		**Value**	
Brier score		0.06	
AUC		0.70	
Calibration in-the-large		-0.03	
Calibration slope		0.89	

Analysis based on multiple imputation using stepwise backwards variable selection with a p-value cut-off of 0.15.

The value of performance criteria was corrected for optimism by cross-validation.

OR: Odds Ratio, 95% CI: 95% Confidence Interval, AUC: Area Under the ROC Curve.

**Fig 2:**
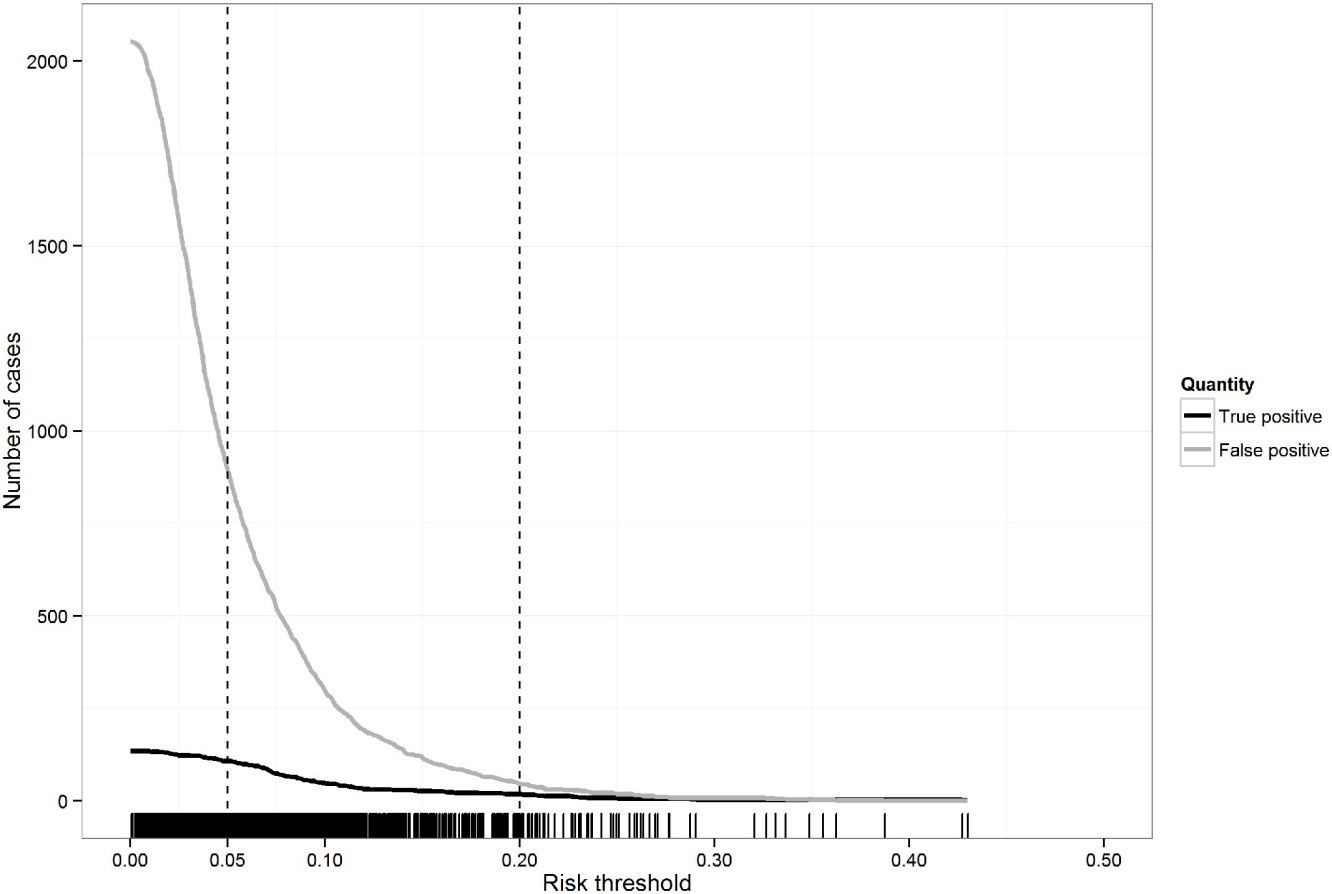
The number of true positive and false positive cases for predictions based on the reduced logistic model on the original dataset using different risk thresholds for classification. Rugs at the bottom correspond to the distribution of predicted risks. The two vertical lines correspond to risk thresholds of 5% and 20%. Results are for the complete-case analysis.

The predicted risk of DSS in study participants based on the reduced model was left skewed with a median (IQR) risk of 4.6% (2.6%-8.0%). Fig 2 displays the number of true positive and false positive cases depending on the chosen risk threshold for classifying subjects as likely to progress to DSS or not. For a low risk threshold, the number of false positive cases was quite high. For example, at a risk threshold of 5%, 108/134 (81%) of cases with DSS would be correctly classified; however, the number of false positive cases would be 8 times higher (894 cases). For a higher risk threshold, the number of false positive cases decreased at the cost of missing true positive cases. For example, at a risk threshold of 20%, although there were only 46 false positive cases only 17/134 (13%) of the cases with DSS could be detected by the model.

### Potential value of daily haematocrit levels and platelet counts

**Fig 3:**
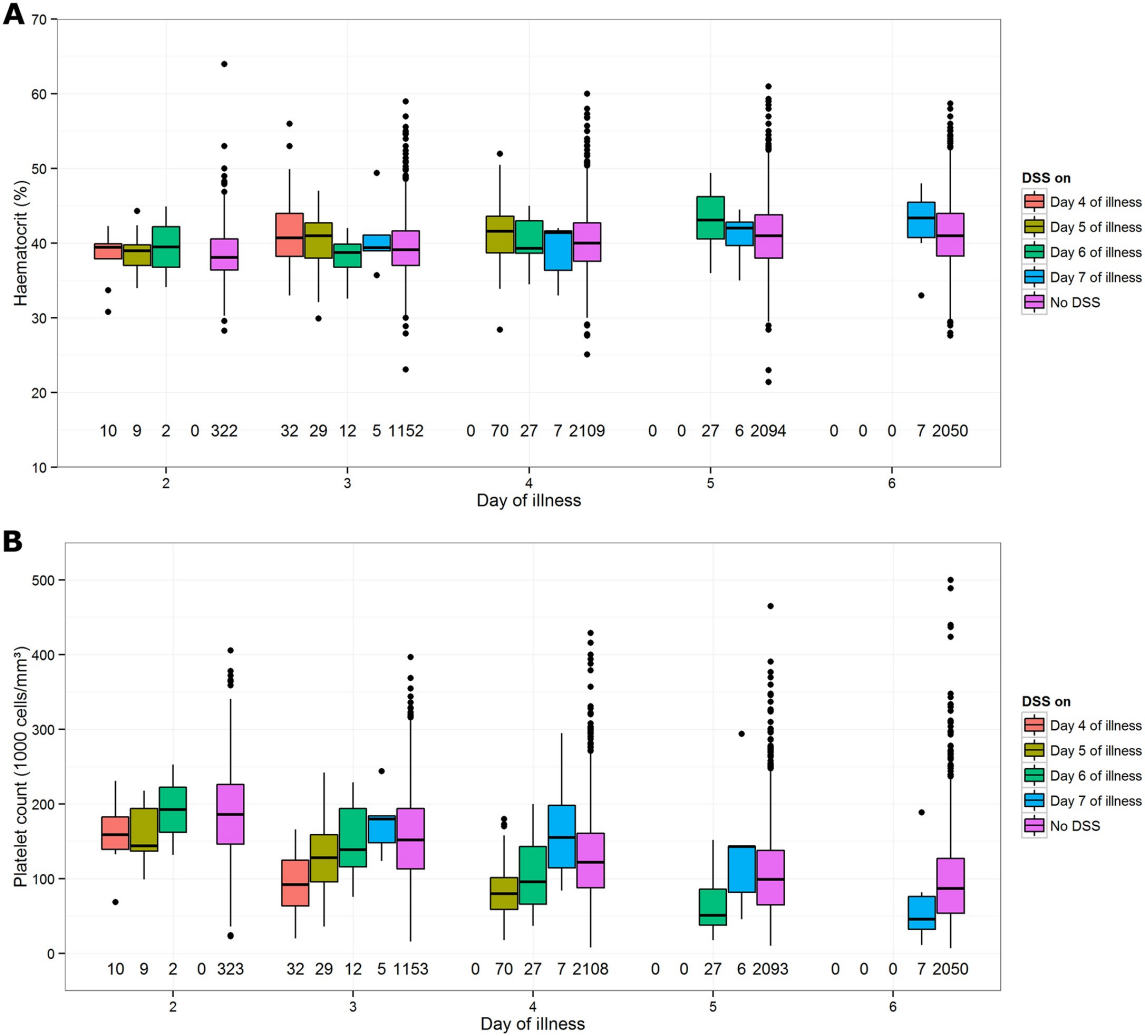
Box plots describing changes in haematocrit (Panel A) and platelet (Panel B) values from day 2 to day 6 of illness, among study participants who developed DSS between days 4 and 7 of illness, as well as participants who never developed DSS. The graph includes all relevant patients who had at least one platelet count recorded between days 2 and 6 of illness, apart from one extreme outlier (without DSS) in whom a platelet count of 779,000 cells/mm^3^ was recorded on day 5. The numbers displayed at the bottom of each panel represent the number of patients contributing to each box plot.

**Fig 4:**
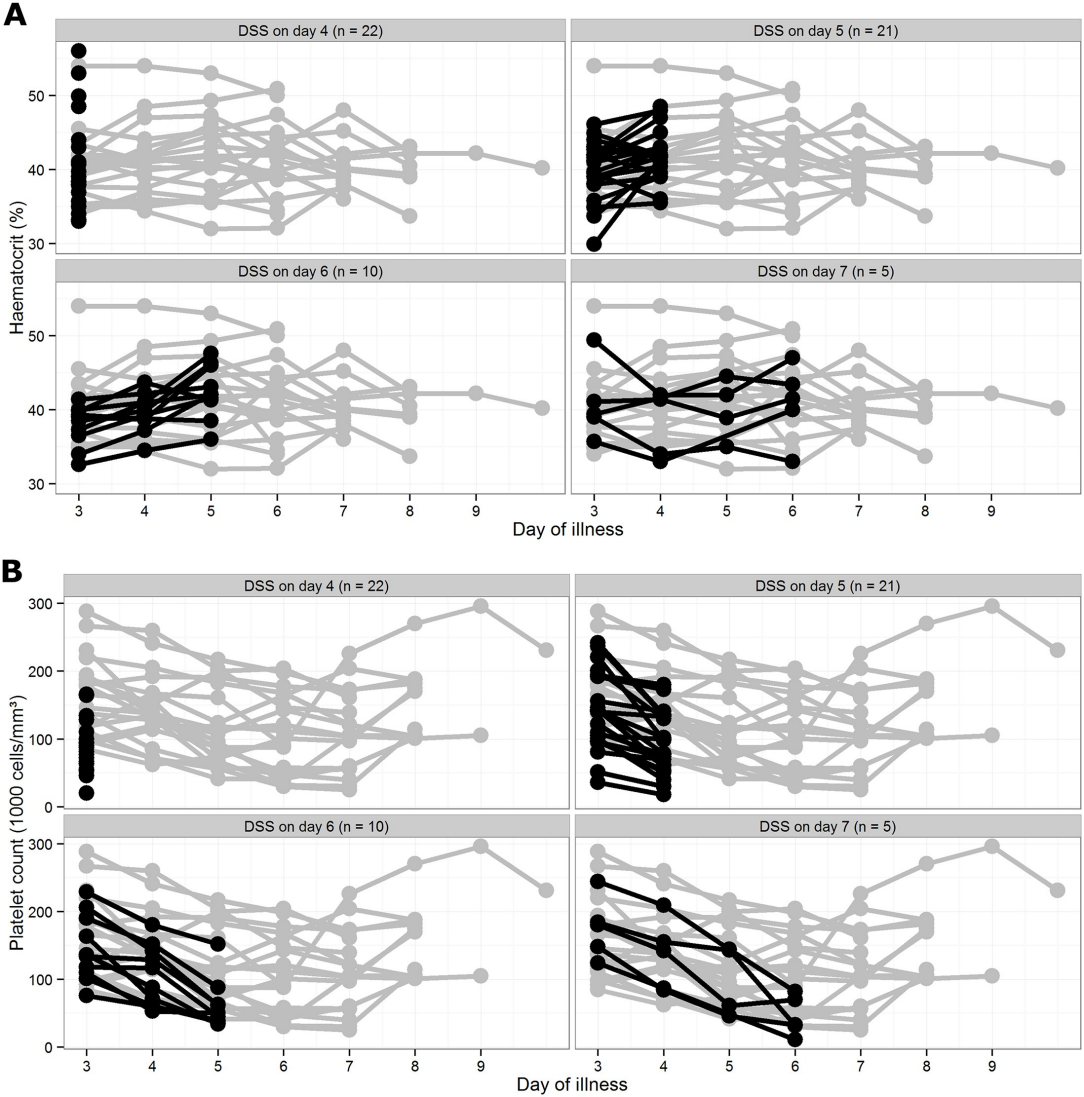
Trajectories of longitudinal haematocrit (Panel A) and platelet (Panel B) values for patients enrolled on day 3 who developed DSS between days 4 and 7 of illness (black lines and dots, with data censored from the day DSS occurred) and a control group of 20 randomly chosen patients enrolled on day 3 who did not develop DSS (grey lines and dots). For the haematocrit levels the black and grey symbols appear randomly superimposed, while for the platelet counts, the black dots tend to be generally lower than their grey counterparts, especially on the day before development of shock—i.e. patients with DSS tend to have lower platelet counts than patients without DSS on a specific day of illness, with the largest difference apparent on the day before shock occurs.

In agreement with the findings of the baseline prognostic models, no clear difference in daily haematocrit levels was apparent between study participants who did and did not develop DSS (Fig 3, Panel A). By contrast, superimposed on the progressive general reduction in platelet counts observed (as expected), in all participants between days 2 and 6 of illness, Panel B demonstrates that platelet counts in the patients who developed DSS tended to be lower than in patients who never progressed to DSS, and that this difference was most pronounced on the day before DSS occurred (Fig 3). This trend is also apparent in the plots of individual trajectories of haematocrit and platelet count when restricted to participants enrolled on day 3 of illness (Fig 4). This observation highlights the fact that while a daily platelet count can be helpful for predicting likely progression, the prognostic relevance is time-limited; thus today’s platelet value is useful in assessing the risk for DSS within the next 24 hours, but may not be very informative in predicting development of DSS in two or more days time.

Table 4 also shows clear negative relationships between sequential platelet data and the occurrence of DSS: the risk of subsequent DSS decreased for patients with higher current platelet counts and lower relative decreases in platelet counts compared to the previous day’s value. In other words, subjects with a low current platelet count and/or a pronounced decline compared to the previous day’s value are at a higher risk of DSS. Importantly, these relationships persisted on days 3, 4, and 5 of illness (odds ratio of 0.91, 0.89, and 0.83 for each 10,000 cell/mm^3^ higher current platelet count, on days 3, 4, and 5, respectively). Moreover, prediction models including updated platelet counts demonstrated better ability to discriminate patients who developed DSS from others, compared with the respective baseline prediction model when applied on day 4 or 5 of illness (AUC of 0.72–0.74 for updated models vs. 0.66 for baseline model on day 4, and 0.67–0.73 vs. 0.51 on day 5). Similar analysis with daily haematocrit levels did not suggest any potential predictive value (Table S5 in S1 Appendix).

**Table 4:** Estimated adjusted effects of platelet count at enrolment (baseline value) and ensuing serial platelet data (current value, % change from previous day) for each day of illness, on the subsequent development of DSS. Associated discrimination (AUC) is also shown.

	Day 3 of illness		Day 4 of illness		Day 5 of illness	
Number of events/sample size	59/877 (7%)		37/848 (4%)		15/823 (2%)	
	OR (95% CI)	AUC	OR (95% CI)	AUC	OR (95% CI)	AUC
Baseline value, i.e. value on day 3 of illness [+ 10,000 cells/mm^3^]	0.91 (0.86, 0.96)	0.68	0.97 (0.91, 1.03)	0.66	1.01 (0.92, 1.10)	0.51
Current value [+ 10,000 cells/mm^3^]	0.91 (0.86, 0.96)	0.68	0.89 (0.83, 0.95)	0.72	0.83 (0.71, 0.94)	0.67
% change from previous day [+ 10%]	-	-	0.72 (0.61, 0.84)	0.74	0.68 (0.54, 0.85)	0.73

These analyses were performed on patients enrolled on day 3 of illness only.

For each day of illness, the sample size refers to the number of patients still at risk on that day (i.e. those without DSS up to that day), and the number of events refers to the number of patients who developed DSS on any subsequent day.

Each analysis is based on a logistic regression model with the development of DSS on subsequent days (yes/no) as the outcome, the specific aspect of platelet count dynamics (baseline or current value, or % change) as the main covariate and other predictors from the reduced logistic regression model at baseline (age, sex, history of vomiting, temperature, palpable liver) as additional covariates.

OR: Odds Ratio, 95% CI: 95% Confidence Interval, AUC: Area Under the ROC Curve (corrected for optimism by cross-validation).

## Discussion

Dengue is a viral infection of increasing global significance that evolves rapidly over a short time-course and displays a wide range of clinical manifestations. Although much has been written from an empirical standpoint about the clinical spectrum of disease few formal descriptions based on prospectively collected data have been published; given the highly variable disease evolution this may reflect the need for enrolment of substantial case numbers to allow meaningful interpretation of the relevance of different clinical events. We present a detailed description of the clinical features of dengue observed in more than 2000 Vietnamese children enrolled within 4 days of fever onset, of whom 143 (6%) went on to develop DSS. Using this unique dataset we were able to identify several baseline factors (male sex, day of illness at enrolment, history of vomiting, body temperature, palpable liver, platelet count) that were associated with subsequent development of DSS, as well as to investigate dynamic aspects of serial platelet and haematocrit changes for risk prediction. While the prediction models based on baseline information demonstrated only moderate performance and limited clinical utility, incorporating features of longitudinal platelet count kinetics into the models improved their performance.

An important feature of this study is that the study population was enrolled at an early stage of the illness, i.e. all participants were enrolled within the first 4 days after fever onset when they had relatively mild disease. Although the concept of hospitalization based on warning signs has become established in recent years, the strong community perception of dengue as a dangerous disease in childhood, combined with major transport/logistic difficulties during the study years especially at night, resulted in a low threshold for hospitalization at that time. Early enrolment likely explains the lower incidence of DSS in our cohort compared to contemporaneous reports from Ho Chi Minh city that described all hospitalized cases [12]. The finding of a significant association between male gender and a higher risk for DSS, which contrasts with the evidence from previous epidemiological studies [29,30], was somewhat surprising and may also relate to early enrolment. In the cohort of children directly admitted to PICU with DSS during the same time period [6], females were more likely to be admitted to hospital on the day of DSS than males (49% of females compared to 41% of males, chi-squared test p<0.001). If severe females presented to hospital later than their male counterparts, this could lead to an underrepresentation of severe females in our study population. Of note, a general male-bias in paediatric admissions has been noted previously, potentially explained by differences in parental health-seeking behaviour, or by true differences in biological characteristics and/or disease susceptibility between genders [12,42–44]. Even though males were significantly heavier than females (with adjustment for age, Table S6 in S1 Appendix), it is unlikely that the unexpected association between male gender and higher risk of developing DSS can be explained by weight differences since a) weight by itself was not a risk factor for DSS, and b) weight was already adjusted for in the multivariable analysis of risk factors for DSS.

Interestingly, we did not find evidence of a relationship between haematocrit and risk of progression to DSS, even though haematocrit but not platelet count was a strong predictor of severe outcome in our study of children with established DSS [21]. Thus the platelet count and haematocrit seem to be relevant for risk prediction during different stages of the disease. We found the absolute platelet count on a given day during the febrile phase to be an important risk factor for developing DSS, and furthermore that changes in the platelet count over time are also related to changes in the likelihood of developing DSS. As the main underlying pathophysiological abnormality in DSS is plasma leakage [3], these findings suggest a potential role for platelets in the induction of plasma leakage, a phenomenon supported by the recent work by Hottz et al. [45]. Haematocrit levels, by contrast, likely reflect the extent of plasma leakage balanced by a variety of compensatory mechanisms. Haematocrit may be less important in the early phase of disease, as found in this study, because plasma leakage is less pronounced in this stage. However, once a patient has progressed to established DSS, increasing haemoconcentration becomes an important indicator of ongoing or profound leakage. Haematocrit changes are typically monitored every few hours when shock is anticipated and might have proved useful for short-term risk prediction (hours rather than days), but we did not have access to these data. In any case complex modeling would be needed to incorporate such data into an algorithm and a predictive window of less than 24 hours is less relevant from the public health perspective.

Several features that we identified at study enrolment—the absolute platelet count, vomiting and a palpable liver—have already been reported in the published literature as predictors of DSS [10,29]. We also identified a higher baseline temperature to be independently associated with an elevated risk of DSS, a finding which might be explained by the positive correlation between fever and viral load [46,47]. An association between enrolment at an earlier day of illness and a higher risk of DSS was also found, but only in the multivariable not the univariate analyses; this is likely to be an artifact attributable to adjustment for other clinical features assessed at enrolment, notably the platelet count, in the multivariable analyses. By adjusting for platelet count, the reported odds ratio corresponds to the comparison of two subjects who were enrolled on two consecutive days of illness but had the same platelet count on their respective day of enrolment. As platelet counts decrease over time during the illness course [48], the subject enrolled earlier would have a lower platelet count relative to their day of enrolment and, as platelet count is strongly inversely associated with the risk of DSS development, this might explain the reported effect.

The baseline prediction model presented here has an AUC of 0.70 and good calibration on internal validation. Unfortunately this moderate performance, combined with the low incidence of DSS, results in a model of only limited clinical utility. To be useful in clinical practice, a prediction model should be able to correctly identify most subjects who subsequently develop DSS. However, as illustrated in Fig 2, this would mandate a very low risk threshold and the number of true positives would be swamped by the much larger number of false positives. A prediction rule with a high false positive rate implies a significant additional patient burden for high dependency and/or intensive care units to which such cases might be transferred, and this may negatively affect the quality of care that patients who are truly at high risk of DSS receive, especially in endemic countries with scarce resources. The fact that this model was carefully developed using data from over 2300 children hospitalised during the febrile phase of dengue suggests that reliance on readily available baseline characteristics and warning signs is not sufficient for reliable risk prediction for DSS, and that incorporation of novel markers with higher predictive value is likely to be required to achieve better models. In addition to virological, immunological and host genetic factors, novel markers that represent or better characterise microvascular dysfunction and/or intravascular volume status are potential candidates [30]. However, their value still needs to be verified in well-designed prospective studies. Additionally, for practical application in resource-limited settings, inexpensive but reliable systems to measure novel biomarkers that prove to be robust predictors would need to be developed and made widely accessible [30,49], highlighting the current need to capitalise efficiently on parameters that are already available routinely in many centers.

Another issue that could affect the applicability of such baseline prediction models, is the lack of transferability of the baseline prediction to later time-points [50,51]. In our study, the predictive performance of the baseline platelet count and other baseline risk factors completely deteriorated (AUC = 0.51) when aiming to predict DSS occurring more than 2 days later. In contrast, by incorporating dynamic aspects of the platelet count kinetics (i.e. the current/updated value or the % change from the previous day), the models remained relevant to DSS prediction at later time-points. Similar findings have been reported for longitudinal biomarkers in other diseases [20,52–55]. The implication of this finding is twofold: first, since the predictive value of a particular platelet count is limited to one day ahead, the necessity to monitor the platelet count daily during the febrile phase is emphasized; second, the potential role for a dynamically updated prediction model to improve triage of dengue patients is suggested. Ideally, such a model would be incorporated into a simple algorithm that could be used by clinicians in endemic areas to monitor patients on a daily basis, adaptively suggesting the appropriate level of observation and/or treatment as new information is collected. For the present however, in settings where measurement of platelet count is not yet available, for example in peripheral facilities in endemic countries with low-resources, it would be beneficial to make this important and simple biomarker accessible to clinicians in order to improve patient triage and management.

Unfortunately we were not able to include other potentially relevant risk factors, for example sequential white blood cell counts, as this information was not routinely recorded in the study files at the time the original study was conducted; however it would be interesting to incorporate such information into prospective studies assessing the utility of dynamic prediction modeling for dengue. Another limitation of the present dataset is that the sample size and the number of DSS cases included in the analysis of longitudinal platelet counts, which was restricted to the 908 patients enrolled on day 3 of illness, was too small to draw definite conclusions. This also prevented exploration of more complex models with time-varying coefficients for the longitudinal platelet counts, or assessment of non-linear platelet effects. Moreover, the current study was restricted to hospitalized patients admitted to a single hospital in Vietnam, and the longitudinal data available was limited to daily hematocrit and platelet values. To develop reliable dynamic prediction models, even larger and richer datasets which collect detailed longitudinal information on several candidate risk factors will be required, ideally involving a range of clinical settings in dengue endemic areas [49].

In conclusion, this study has confirmed the value of a number of established risk factors for DSS among children with dengue, and has demonstrated that prediction can be improved by dynamically incorporating sequential platelet values into the models. Although the study was performed among hospitalized children, the structure of the healthcare and transport systems in operation in Ho Chi Minh City at that time resulted in early admission of many children with possible dengue, and the findings may be applicable to the population of children now managed as outpatients during the early phase of their illness in many large cities across southeast Asia. The findings reinforce the view that in the early febrile phase dengue is typically a rather non-specific illness, but also provide strong support for the WHO recommendation to perform daily full blood counts in order to monitor the platelet count closely in these patients [56]. The next step would be to initiate large outpatient based research programs aimed at developing and validating more complex dynamic models which could improve identification and management of cases likely to develop DSS in the early febrile phase, as well as assessing the utility of warning signs that are currently recommended for risk prediction during the transition to the critical phase. Further methodological research on how to build and assess dynamic prediction models in acute infectious diseases will be important to achieve these goals, alongside efforts to acquire and make available suitably rich datasets.

## Supporting information

S1 Checklist STROBE checklist. (DOC)

S1 Appendix Detailed information on clinical outcome, pre-defined candidate predictors, and development of the baseline prognostic model, Table S1-S6. (DOCX)

S2 Appendix Observational data of 2301 children with dengue infection in the cohort. (ZIP)
