# Supplementary material for: The value of daily platelet counts for predicting dengue shock syndrome: Results from a prospective observational study of 2301 Vietnamese children with dengue
Source: PLoS Negl Trop Dis. 2017 Apr 27;11(4):e0005498. doi: 10.1371/journal.pntd.0005498 (PMC5407568; doi:10.1371/journal.pntd.0005498)
Supplement: S1 Appendix — (DOCX) [file pntd.0005498.s002.docx]

Appendix

## Clinical outcome and pre-defined candidate predictors

##### **Table S1. Clinical outcome and pre-defined candidate predictors.**

| **Variable** | **Unit or possible values** | **Type** |
| --- | --- | --- |
| ***Clinical outcome*** |  |  |
| Dengue shock syndrome | Yes/No | Binary |
| ***Candidate predictors at enrolment*** |  |  |
| Age | Year | Continuous |
| Sex | Female/Male | Binary |
| Weight | Kg | Continuous |
| Day of illness | Day of illness at enrolment | Continuous |
| History of tiredness | Yes/No | Binary |
| History of vomiting | Yes/No | Binary |
| Tourniquet test | Negative/Equivocal/Positive | Categorical |
| Temperature | Body temperature [°C] measured in the axilla | Continuous |
| Pulse rate | Beats per minute | Continuous |
| Systolic blood pressure | mmHg | Continuous |
| Mucosal bleeding | Yes/No | Binary |
| Abdominal pain | Yes/No | Binary |
| Palpable liver | Yes/No | Binary |
| Haematocrit | Haematocrit value [%] | Continuous |
| Platelet count | Platelet count [cells per mm³] | Continuous |
| Serotype | Serotype of dengue virus based on PCR: DENV-1, DENV-2, DENV-3, DENV-4, Mixed, or Negative | Categorical |
| Immune status | Immune status of patient based on serology: probable primary dengue, probable secondary dengue, and unclassifiable status | Categorical |
| ***Daily haematocrit level and platelet count*** | |  |
| Current value | Value at day of assessment | Continuous |
| % increase from previous day | Calculated by 100 x (current value - value on previous day)/value on previous day [%] | Continuous |

Dengue shock syndrome was defined based on the WHO guidelines 2009.

## Treatment of missing data

Multivariate Imputation by Chained Equations (MICE) as implemented in the R package mice version 2.22 [1] was used to generate multiple imputed data sets based on a set of imputation models (each variable with missing values has one imputation model). In this study, the chosen imputation models were predictive mean matching for continuous variables, logistic regression for binary variables and multinomial regression for categorical variables with more than 2 classes. As recommended, these models included outcomes and all candidate predictors (with only linear terms for continuous covariates and no interaction terms) [2]. The visit order of the variables in each iteration cycle was according to their (increasing) number of missing values. In total twenty imputed datasets were created and 50 cycles per dataset were performed. Of notes, this exceeds the minimum required numbers of imputed datasets and repeated cycles according to current recommendation which are 5 (the percentage of incomplete cases) and 10-20, respectively [2].

## Statistical models for baseline prediction

Statistical models chosen in this study included logistic regression as the main model and alternative statistical models including the lasso, generalized additive models (GAM), classification and regression tree (CART), and gradient boosting with trees as base learners [3]. While the validity of the logistic regression models is based on the assumptions of linearity and additivity of covariate effects, these assumptions are relaxed in several of alternative approaches. Therefore, these models acted as comparators in order to detect any defects in the main logistic model.

Parameters of logistic regression models were estimated using standard maximum likelihood estimation. Estimation of the penalty parameter for the lasso was based on standardized covariates and leave-one-out cross validation with the likelihood as the optimization criterion as implemented in the R package glmnet version 2.0.2 [4]. The CART model built and pruned back a classification tree using default parameter settings of the R package rpart version 4.1.9 [5]. The GAM model was built based on default settings of the R package mgcv version 1.8.6 [6]. The implementation automatically estimates the degrees of freedom of smooth terms based on generalized cross-validation. To fit a "pure" additive model, the interaction terms were not included in the model formula. Finally, a generalized boosted regression model with a Bernoulli distribution for the outcome was fitted using classification trees as base learners as implemented in the R package gbm version 2.1.1 [7]. Each tree has a depth of at most 2 which allows for 2-way interactions. The number of 3000 iterations and the learning rate of 0.001 were chosen as recommended by the gbm package author.

In multiple imputation analysis, estimates and asymptotic covariance matrices (and associated Wald-type tests) were combined across multiple imputed datasets using Rubin's rule and likelihood ratio tests for multiple imputed datasets were calculated using the method of Meng and Rubin [2,8].

## Linearity and additivity assessment

The validity of the logistic regression models is based on the assumptions of linearity and additivity of covariate effects. Based on an initial prediction model including all candidate predictors as linear and additive terms, we assessed linearity of all continuous variable and possible interactions between gender and day of illness at enrolment, gender and all other covariates, and day of illness with all other covariates. If pronounced non-linear or interaction terms were detected during this assessment, they were added to the model.

Specifically, the linearity assumption was assessed in two ways:

- Numerically by performing likelihood ratio test to compare goodness-of-fit between the initial model and a more flexible model which allows for non-linear effect (quadratic functions or natural cubic splines with 4 degrees of freedom).
- Graphically by assessing estimated non-linear effects of each continuous variable on the outcome from a flexible multivariable model which allows for non-linearity. The flexible multivariable model was chosen as a generalized additive model which included all continuous variables of interest modeled as natural cubic spline functions with automated selection of the required degree of smoothness, and the partial effect of each variable on outcome was extracted and visualized using term plots [9].

For additivity, interactions were assessed by overall interaction tests, i.e. likelihood ratio tests comparing the initial model and the extended model which also included pre-defined interaction terms. If this overall test was significant, further investigation was performed to identify the specific interaction.

For multiple-imputation analysis, p-values for linearity and additivity tests were pooled across multiple imputed sets using the method of Meng and Rubin [8].

In this study, the linearity assessment suggested that linear terms were sufficient for all continuous candidate predictors except that there was some indication of non-linearity for the effect of age on the development of DSS, as displayed in Figure S1. However, the displayed non-linearity for age was not strong and all tests did not reach statistical significance. There was also no evidence of any interactions between gender or day of illness with any other variables Table S2. Thus, no non-linear terms or interactions were added to the pre-defined multivariable model.

##### **Table S2. Linearity and additivity tests in the multivariable logistic regression model for the development of DSS using complete-case and multiple imputation analyses.**

|  | **Complete case analysis** | | |  | **Multiple imputation** |
| --- | --- | --- | --- | --- | --- |
|  | **Deviance** | **df** | **p value** |  | **p value** |
| **Linearity tests (compared to a quadratic function)** | | | | | |
| Age | 3.94 | 1 | 0.05 |  | 0.06 |
| Weight | 0.88 | 1 | 0.35 |  | 0.37 |
| Temperature | 0.10 | 1 | 0.75 |  | 0.75 |
| Pulse | 0.35 | 1 | 0.56 |  | 0.52 |
| Systolic blood pressure | 0.01 | 1 | 0.91 |  | 0.76 |
| Haematocrit | 0.50 | 1 | 0.48 |  | 0.50 |
| Platelet count | 1.13 | 1 | 0.29 |  | 0.25 |
| **Linearity tests (compared to a natural cubic spline with 4 degrees of freedom)** | | | | | |
| Age | 5.30 | 3 | 0.15 |  | 0.12 |
| Weight | 6.98 | 3 | 0.07 |  | 0.16 |
| Temperature | 5.12 | 3 | 0.16 |  | 0.14 |
| Pulse | 1.47 | 3 | 0.69 |  | 0.59 |
| Systolic blood pressure | 3.17 | 3 | 0.37 |  | 0.55 |
| Haematocrit | 2.18 | 3 | 0.54 |  | 0.64 |
| Platelet count | 2.90 | 3 | 0.41 |  | 0.39 |
| **Additivity assessment (Interaction tests)** | | | | | |
| Gender vs. others | 12.40 | 15 | 0.65 |  | 0.80 |
| Day of illness vs. others | 13.82 | 15 | 0.54 |  | 0.29 |
| Gender vs. age | 0.01 | 1 | 0.91 |  | 0.87 |


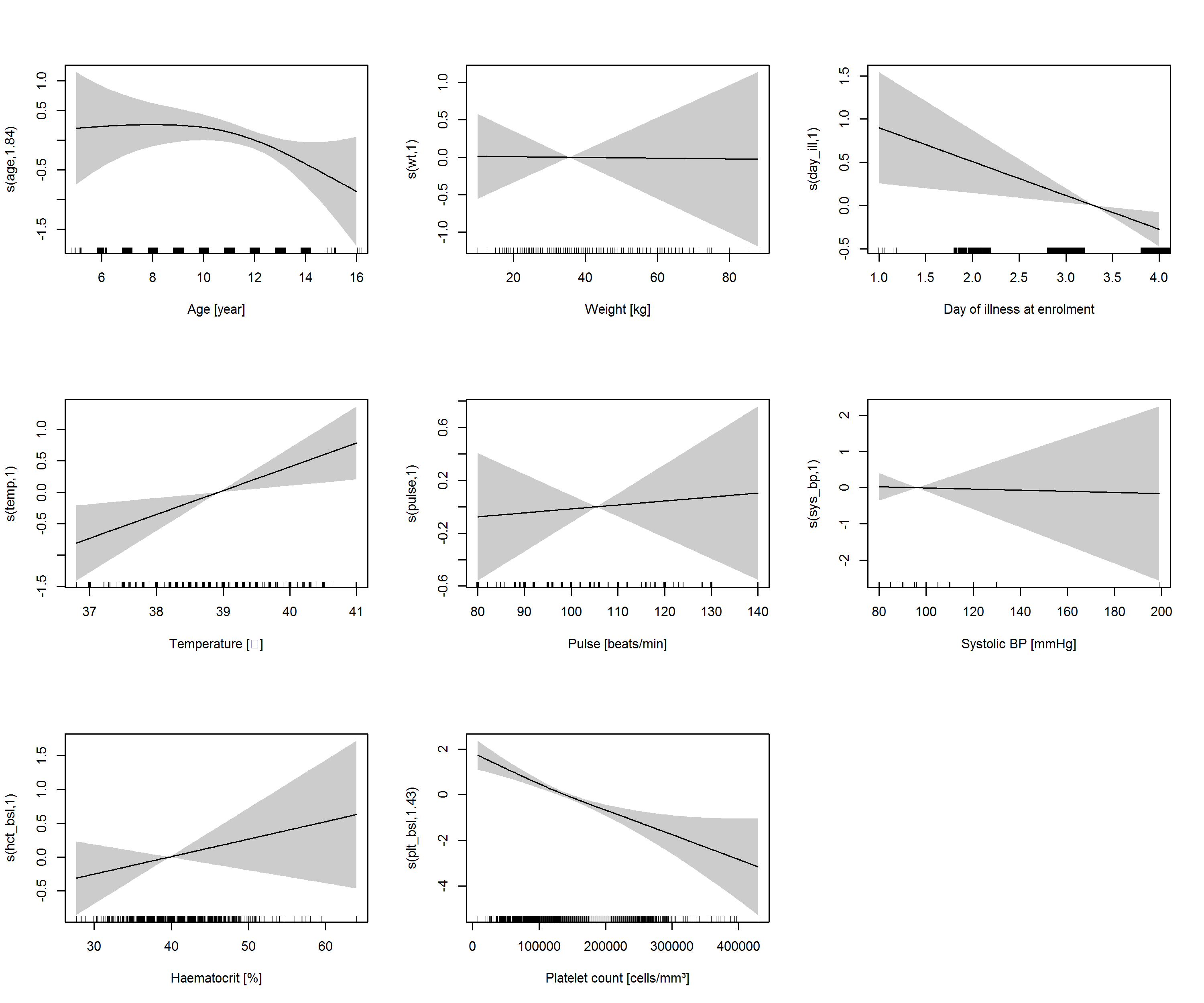


##### **Figure S1. Plots of estimated component smooth functions of a generalized addictive model (GAM) fit for development of DSS with continuous covariates modelled using natural cubic spline functions and integrated smoothness estimation.** Solid lines correspond to spline functions estimated by GAM; grey areas correspond to point-wise 95% confidence intervals of the estimated values.

##### **Table S3. Adjusted effects of candidate predictors on outcome in the full and reduced logistic regression models based on complete cases (N = 2186).**

|  | **Full model** | | |  | **Reduced model** | | |
| --- | --- | --- | --- | --- | --- | --- | --- |
| **Predictor** | **OR** | **(95% CI)** | **p value** |  | **OR** | **(95% CI)** | **p value** |
| Age [+1 year] | 0.92 | (0.83, 1.01) | 0.092 |  | 0.92 | (0.85, 1.00) | 0.04 |
| Sex: Female | 0.64 | (0.43, 0.94) | 0.024 |  | 0.63 | (0.43, 0.92) | 0.02 |
| Weight [+1 kg] | 1.00 | (0.98, 1.02) | 0.91 |  | - | - | - |
| Day of illness at enrolment | 0.67 | (0.51, 0.88) | 0.0044 |  | 0.68 | (0.52, 0.90) | 0.006 |
| History of tiredness: Yes | 0.92 | (0.56, 1.59) | 0.76 |  | - | - | - |
| History of vomiting: Yes | 2.17 | (1.51, 3.14) | <0.001 |  | 2.19 | (1.53, 3.16) | <0.001 |
| Tourniquet test |  |  | 0.46 |  | - | - | - |
| - Negative | 1.00 |  |  |  | - | - | - |
| - Equivocal | 1.05 | (0.64, 1.66) |  |  | - | - | - |
| - Positive | 0.78 | (0.50, 1.21) |  |  | - | - | - |
| Temperature [+1 °C] | 1.45 | (1.10, 1.91) | 0.0076 |  | 1.43 | (1.10, 1.86) | 0.007 |
| Pulse [+10 beats/min] | 1.03 | (0.85, 1.23) | 0.79 |  | - | - | - |
| Systolic blood pressure [+10 mmHg] | 0.99 | (0.77, 1.23) | 0.9 |  | - | - | - |
| Mucosal bleeding: Yes | 1.09 | (0.52, 2.06) | 0.81 |  | - | - | - |
| Abdominal pain: Yes | 1.12 | (0.69, 1.79) | 0.63 |  | - | - | - |
| Palpable liver: Yes | 1.74 | (0.99, 2.98) | 0.055 |  | 1.78 | (1.08, 2.83) | 0.024 |
| Haematocrit [+1 %] | 1.02 | (0.98, 1.07) | 0.28 |  | - | - | - |
| Platelet count [+10,000 cells/mm³] | 0.89 | (0.85, 0.92) | <0.001 |  | 0.89 | (0.85, 0.92) | <0.001 |

*OR: Odds Ratio, 95% CI: 95% Confidence Interval*

## Variable selection

As some candidate predictors may have negligible effects on the outcome and the full model is generally complex, it is necessary to simplify the model before applying it to clinical practice [10].

For complete case and multiple imputation analysis, stepwise backwards selection was used to find the best combination of predictors [3]. For complete case selection was based on the Akaike information criterion (AIC). In multiple imputation analysis, at each variable selection step, the model of interest was fitted to all imputed datasets and the least significant predictor was excluded if its pooled p value was larger than 0.15 (the p-value cut-off of 0.15 was chosen to approximately mimic variable selection based on AIC). The final model was obtained by applying Rubin's rule to aggregate parameter estimates from a model which included all predictors that remained after the variable selection procedure across imputed datasets.

## Assessment of model performance

The performance of developed models was assessed in terms of overall performance, discrimination and calibration. The overall performance of prediction models was quantified with the Brier score which is the average squared difference between patients' observed outcomes (0 for patients without the outcome, 1 for patients with the outcome) and their predicted risks. This quantity can range from 0 for a perfect model to a maximum value depending on the incidence of outcome for a non-informative model (for example 0.25 with a 0.5 incidence of the outcome). Discrimination measures how well a prognostic model can differentiate subjects with and without outcome. This aspect of model performance can be assessed using the c-statistic defined as the AUC. An AUC of 1 indicates perfect discrimination whereas an AUC of 0.5 indicates that the model does not discriminate better than random guessing. Calibration measures the agreement between observed and predicted outcomes. This measure can be quantified in terms of calibration-in-the-large and the calibration slope. Calibration-in-the-large assesses how well the average predicted risk matches the overall observed incidence of the outcome. The optimal value of calibration-in-the-large is 0. Calibration-in-the-large of <0 or >0, respectively, indicate that predicted outcomes are systematically too high or too low. The calibration slope reflects the extremeness of the predicted outcome and is compared to 1. A calibration slope <1 indicates that the predictions are too extreme; whereas a calibration slope >1 implies that the predictions are not extreme enough.

As no independent validation datasets were available to assess performance of prediction models developed in this project, models were evaluated on the development dataset which imposes the risk of optimism, i.e. over-estimation of performance due to over-fitting [10]. In order to compensate for optimism and to get a realistic assessment of the performance of the entire model development process, all performance measures were corrected for optimism using 10-times repeated 10-fold cross-validation technique. Specifically, the whole modeling procedure except for assumption assessments was firstly repeated 10 times by using a selection of nine tenths of the data for model development and one tenth for validation, respectively [10]. The cross-validation was further repeated ten times to minimize dependence on the random split into ten sub-datasets. The performance of the derived model on the test sets was then averaged across the 100 test sets to provide overall optimism-corrected performance measures.

For multiple imputed datasets, the above model validation steps were adjusted according to current recommendation [2]. The whole modeling procedure for each statistical model of interest was applied to each imputed training set. Predictions of each fitted model on the corresponding imputed test set were obtained and then compared to observed outcomes in the test set of each imputed dataset to derive performance measure. These measures were then averaged across imputed test sets to provide a single set of measures for each model.

##### **Table S4. Performance of different baseline prediction models for development of DSS based on multiple imputation and complete-case analyses.**

|  | **Full** | **Reduced** |  |  |  |  |
| --- | --- | --- | --- | --- | --- | --- |
|  | **logistic regression** | **logistic regression** | **Lasso** | **GAM** | **CART** | **Boosting** |
| **Multiple imputation** |  |  |  |  |  |  |
| Brier | 0.06 | 0.06 | 0.06 | 0.06 | 0.06 | 0.06 |
| AUC | 0.69 | 0.70 | 0.69 | 0.68 | 0.57 | 0.68 |
| Calibration in-the-large | -0.03 | -0.03 | -0.03 | -0.03 | -0.03 | -0.11 |
| Calibration slope | 0.81 | 0.89 | 1.04 | 0.75 | 0.57 | 1.07 |
| **Complete-case analysis** |  |  |  |  |  |  |
| Brier | 0.06 | 0.06 | 0.06 | 0.06 | 0.06 | 0.06 |
| AUC | 0.70 | 0.71 | 0.70 | 0.68 | 0.58 | 0.69 |
| Calibration in-the-large | -0.03 | -0.03 | -0.03 | -0.03 | -0.03 | -0.11 |
| Calibration slope | 0.82 | 0.93 | 1.06 | 0.76 | 0.01 | 1.09 |

*Reduced logistic regression used stepwise selection with AIC as criteria to select variables.*

*All values were corrected for optimism by 10-times repeated 10-fold cross-validation.*

*GAM: Generalized Addictive Models, CART: Classification And Regression Tree, AUC: Area Under the ROC Curve**.*

## Potential value of daily haematocrit

##### **Table S5. Estimated adjusted effects of haematocrit levels at enrolment (baseline value) and daily values (current value, % change from previous day) on each day of illness on the subsequent development of DSS. Associated discrimination (AUC) is also shown.**

|  | **Day 3 of illness** | |  | **Day 4 of illness** | |  | **Day 5 of illness** | |
| --- | --- | --- | --- | --- | --- | --- | --- | --- |
| Number of events/sample size | 59/876 (7%) | |  | 37/847 (4%) | |  | 15/822 (2%) | |
|  | **OR (95% CI)** | **AUC** |  | **OR (95% CI)** | **AUC** |  | **OR (95% CI)** | **AUC** |
| Baseline value [+ 1% ] | 1.02 (0.96, 1.09) | 0.68 |  | 1.00 (0.91, 1.09) | 0.65 |  | 0.97 (0.83, 1.11) | 0.50 |
| Current value [+ 1%] | 1.02 (0.96, 1.09) | 0.68 |  | 1.02 (0.93, 1.10) | 0.66 |  | 1.03 (0.92, 1.15) | 0.53 |
| % change from previous day [+ 1%] | - | - |  | 1.01 (0.98, 1.04) | 0.66 |  | 1.03 (0.98, 1.07) | 0.55 |

These analyses were performed on patients enrolled on day 3 of illness only.

On each day of illness, sample size refers to the number of patients still at risk on that day, and the number of events refers to the number of patients who developed DSS on subsequent days.

Each analysis is based on a logistic regression model with the development of DSS on subsequent days (yes/no) as the outcome, the specific aspect of haematocrit levels dynamics (baseline or current value, or % change) as the main covariate and other predictors from the reduced logistic regression model at baseline (age, sex, history of vomiting, temperature, palpable liver, platelet count) as additional covariates.

OR: Odds Ratio, 95% CI: 95% Confidence Interval, AUC: Area Under the ROC Curve (corrected for optimism by cross-validation).

**Relationship between body weight and sex amongst participants**

**Table S6. Relationship between body weight and sex amongst study participants (n = 2301)**

| **Sex** | **Total** | **Available** | **Body weight (kg)** |  | **Coefficient** | **(95% CI)** | **p value** |
| --- | --- | --- | --- | --- | --- | --- | --- |
| Female | 939 | 937 | 33 (26, 40) |  | 0.0 |  |  |
| Male | 1362 | 1359 | 35 (28, 44) |  | 2.5 | (1.8, 3.2) | <0.001 |

This analysis was based on linear regression with adjustment for age.

95% CI: 95% Confidence Interval.

# References

1. Van Buuren S, Groothuis-Oudshoorn K. MICE: Multivariate imputation by chained equations in R. J Stat Softw. 2011;45: 1–67.

2. White IR, Royston P, Wood AM. Multiple imputation using chained equations: Issues and guidance for practice. Stat Med. 2011;30: 377–99. doi:10.1002/sim.4067

3. Hastie TJ, Tibshirani R, Friedman J. The Elements of Statistical Learning. 2nd ed. New York, NY: Springer New York; 2009. doi:10.1007/b94608

4. Friedman J, Hastie TJ, Tibshirani R. Regularization Paths for Generalized Linear Models via Coordinate Descent. J Stat Softw. 2010;33: 1–22.

5. Therneau T, Atkinson B, Ripley B. rpart: Recursive Partitioning and Regression Trees [Internet]. 2014. Available: http://cran.r-project.org/package=rpart

6. Wood S. Fast stable restricted maximum likelihood and marginal likelihood estimation of semiparametric generalized linear models. J R Stat Soc Ser B. 2011;73: 3–36. doi:10.1111/j.1467-9868.2010.00749.x

7. Greg Ridgeway with contributions from others. gbm: Generalized Boosted Regression Models [Internet]. 2014. Available: https://github.com/harrysouthworth/gbm

8. Meng X-L, Rubin DB. Performing Likelihood Ratio Tests with Multiply-Imputed Data Sets. Biometrika. 1992;79: 103–11. doi:10.2307/2337151

9. Wood S. Generalized Additive Models: An Introduction with R. 1st ed. Chapman and Hall/CRC; 2006.

10. Steyerberg EW. Clinical Prediction Models: a practical approach to development, validation, and updating. New York: Springer; 2010.
